# Fis Is Essential for the Stability of Linear Plasmid pBSSB1 and Affects the Motility of *Salmonella enterica* Serovar Typhi

**DOI:** 10.1371/journal.pone.0037462

**Published:** 2012-07-23

**Authors:** Haifang Zhang, Bin Ni, Xin Zhao, Isaac Dadzie, Hong Du, Qiang Wang, Huaxi Xu, Xinxiang Huang

**Affiliations:** 1 Department of Biochemistry and Molecular Biology, School of Medical Technology, Jiangsu University, Zhenjiang, Jiangsu, China; 2 State Key Laboratory of Pharmaceutical Biotechnology, Department of Biology, Nanjing University, Nanjing, Jiangsu, China; University of Osnabrueck, Germany

## Abstract

pBSSB1 is a 27 kb non-bacteriophage-related linear plasmid first found in *Salmonella enterica* serovar Typhi (*S.* Typhi), but the mechanism underlying the replication of pBSSB1 is currently unknown. Previous reports showed that the factor for inversion stimulation (Fis) encoded by *fis* can affect the replication, transcription and other processes through binding DNA. Here, a *fis* deletion mutant of *S.* Typhi (Δ*fis*) was prepared through the homologous recombination mediated by suicide plasmid and the loss of pBSSB1 in Δ*fis* was observed surprisingly by pulsed field gel electrophoresis (PFGE). Subsequently, the loss of pBSSB1 was verified by PCR and Southern blot. In addition, the motility of Δ*fis* was deficient and the flagellin of Δ*fis* could not be detected by 2-dimensional polyacrylamide gel electrophoresis. All these results show that Fis is essential for the stability of pBSSB1 and affects the motility of *S.* Typhi.

## Introduction

The factor for inversion stimulation (Fis) encoded by *fis* is a small DNA-bending nucleotide-associated protein which plays a role in the transcriptional regulation of a number of genes in diverse bacterial species [1]. Fis was found initially as a co-factor of the site-specific recombination system. It was reported that Fis of *E. coli* is composed of two similar subunits and each subunit consists of 98 amino acids. In the structure of Fis, there is a typical α-helix-turn-α-helix (helix-turn-helix, HTH) domain which could bind to the major groove of the DNA double helix [2]. Fis has wide regulatory roles, such as regulating the bacterial growth, virulence, and flagellum [3]. In addition, Fis can change the structure of bacterial nucleic acid and affect the replication, transcription and other processes through binding the DNA [4].

Plasmid is an extra chromosomal, self replicating genetic element which in many cases is circular. In 1979, the first linear plasmid of prokaryote was found in *Streptomyces rochei* which could produce antibiotics [5]. So far linear plasmids have been found in about a dozen of *Streptomyces*, and the molecular size of these linear plasmids is between 12–640 kb [6]. Subsequently, another kind of linear plasmids were also found in *Borrelia* [7]. In 2007, a linear plasmid named pBSSB1 was reported to be present in *Salmonella enterica* serovar Typhi (*S.* Typhi) z66-positive strain by Baker *et al* [8]. pBSSB1, which is about 27kb-sized, is the first non-bacteriophage-related linear plasmid found in Enterobacteriaceae, and it mediates the unidirectional flagellar phase variation of *S.* Typhi z66-positive strain [8], [9]. However, the mechanism underlying the replication of pBSSB1 is currently unknown.

The protein DnaA, which recognizes the origin of replication *oriC*, is essential for the DNA replication of Bacteria. With the help of DnaC protein, DnaB, a helicase can bind to *oriC* region to open the DNA double helix so as to initiate replication. Results of *in vitro* research show that Fis competes with DnaA protein for the origin of replication, *oriC* to affect DNA replication in *E. coli* [10], [11]. Besides, Fis can bind to the transcription start site of *dnaA* operon to suppress its expression [12]. *dnaA* operon is composed of *dnaA*, *dnaN* and *recF,* coding for the DnaA protein which can recognize the origin of replication *oriC*, β subunit of DNA polymerase III which is responsible for the extension of the newly-replicated DNA chains and the RecF protein which is involved in the recombination and the repair of DNA, respectively [12], [13]. In light of these functions of Fis, we hypothesized that Fis may play a very important role in the replication of pBSSB1.

In this study, we prepared a *fis* deletion mutant of *S.* Typhi (Δ*fis*) through the homologous recombination mediated by suicide plasmid, and performed pulsed field gel electrophoresis (PFGE) to investigate the genome structure of Δ*fis*. It was surprisingly found that the 27 kb linear plasmid has disappeared in Δ*fis*. The loss of linear plasmid in Δ*fis* was verified by PCR and Southern blot. Moreover, only the complementary strain Δ*fis*(pBAD*fis*) can host this linear plasmid while the Δ*fis* and Δ*fis*(pBAD) cannot. These results suggest that *fis* is essential for the stability of plasmid pBSSB1. Since it was previously reported that the gene *fljB* ^z66^ located on pBSSB1 encodes the flagellum and is responsible for the motility of *S.* Typhi z66-positive strain [8], the motility and flagellin of Δ*fis* was examined by semi-solid agar plates and two-dimensional polyacrylamide gel electrophoresis respectively. The results show that the motility of Δ*fis* was deficient and the flagellin of Δ*fis* could not be detected.

## Materials and Methods

### Bacterial Strains and Plasmids

*S*. Typhi GIFU10007, a z66-positive wild-type strain was used in this study. Mutants and plasmids used in this work are listed in Table 1.

**Table 1:** Strains and plasmids used in the present study.

Strain or plasmid	Relevant characteristics	Reference or source
Strains		
* S*. Typhi GIFU10007	wild-type strain; z66^+^	14
SY372λpir	*E. coli* host strain of suicide plasmid	Laboratory collection
Δ*fis*	GIFU10007(Δ*fis*); z66^-^	This work
Δ*fis* (pBAD)	Δ*fis* containing pBAD empety vector	This work
Δ*fis* (pBAD*fis*)	Δ*fis* containing pBAD*fis* recombinant plasmid	This work
* S*. Typhi GIFU10007-1	GIFU10007 containing pBSSB2; Kana^r^	This work
Δ*fis*(pBAD*fis*)(pBSSB2)	Δ*fis*(pBAD*fis*) containing pBSSB2; Kana^r^	This work
Plasmids		
pGMB151	suicide plasmid; *sacB*; Amp^r^	14
pGMB*fis*	pGMB151 containing *fis*	This work
pBAD/gIII	Expression vector; Amp^r^	15
pBAD*fis*	pBAD containing *fis*	This work
pET-28a-c(+)	Kana^r^	Laboratory collection
pKD46	Red helper plasmid; Amp^r^	Laboratory collection
pBSSB2	pBSSB1 containing a kanamycin resistance gene	This work

### Construction of the *fis* Deletion Mutant of *S.* Typhi

Primers used in this study are listed in Table 2. To generate the Δ*fis*, primer pairs F1A/B and F2A/B were used to amplify the fragments F1 (499-bp) and F2 (314-bp) located upstream and downstream of the gene *fis*, respectively. A *BamH*I site was added to the 5′-termini of primers F1A and F2B, and a *Sal*I site was added to the 5′-termini of primers F1B and F2A. Two fragments F1 and F2 were amplified from S. Typhi GIFU10007 and digested with *Sal* I and ligated with DNA Ligation Kit Ver.2 (TaKaRa) to form the homologous fragment, in which 159-bp of the gene *fis* was absent. The fragment was then inserted into the *BamH* I site of the suicide plasmid pGMB151, which carries a sucrose-sensitivity gene *sacB*. The suicide plasmid carrying the deletion of *fis* gene was transferred into wild-type strain by electroporation as previously described [14], [15]. The mutant strain was selected by PCR with primers F1A and F2B. Finally, the selected candidate of the *fis* deletion mutant was confirmed by sequencing analysis and designated as Δ*fis*.

**Table 2:** Primers used in this study.

Primers	Sequence(5′-)	Purpose
F1A(*BamH* I)	AG*GGATCC*GGCAGTTAAGCAGAAAGT	*fis* mutant construction
F1B(*Sal* I)	CT*GTCGAC*GTTACCTGATCCTGAGAGTT	
F2A(*Sal* I)	TAT*GTCGAC*TGCTGCTCTGATGATGG	
F2B(*BamH* I)	TCA*GGATCC*CACCATACCGTCGAAAT	
P-*fis*-A(*Nco* I)	TA*CCATGG*ATACGCTATTGAGGACGC	Complementary expressionof *fis* in Δ*fis*
P-*fis*-B(*Sal* I)	CT*GTCGAC*TTAGTTCATGCCGTATT	
Pa(23173)	TAACAGATAGCCACACACAGT	Confirmation of the presenceor absence of pBSSB1 by PCR
Pb(23750)	TCAAGGAAGACTGAGATTTGT	
P-Kana-A	AATTGATAAAGGAAAGTGGTTCCGTTATAAAAATGGCTTATTCGATATAAGGTCTGACGCTCAGTGGA	Insertion of kanamycin cassettewithin pBSSB1
P-Kana-B	TAGTGGCTCAAAGAGTATTAGAAATTGACAGAGAAAAGAAAGCAGAATGATTTCGGCCTATTGGTTAA	
ORF1-A	GAGAAGATGCCCGTAA	Confirmation of the insertionof kanamycin cassette withinpBSSB1 by PCR
Kana-B	ATGGCTCATAACACCC	

### Pulsed-field Gel Electrophoresis (PFGE)

A single colony of S. Typhi wild strain and mutant strain Δ*fis* was inoculated into 4 ml LB, and cultured overnight with shaking (250 r/min) at 37°C. Bacteria were collected by centrifugation (4000 r/min, 10 min, 4°C) and washed three times with buffer PIV (10 mmol/L Tris, 1 mol/L NaCl, pH7.6). The pelleted bacteria were resuspended in 1 ml buffer PIV and then mixed with 2% low melting agarose gel to make the cell plugs for PFGE. The cell plugs were digested with the fresh lysis buffer (6 mmol/L Tris, 0.1 molL EDTA, 1 mol/L NaCl, 0.5% Brij-58, 0.2% sodium deoxycholate, 0.5% SDS, RNaseA 20 µg/ml, lysozyme enzyme 1 mg/ml) overnight at 37°C. After being washed with buffer ES (0.5 mol/L EDTA, 1% SDS), the cell plugs were digested with buffer ESP (including protease K 100 µg/ml of the ES) overnight at 50°C to digest bacterial protein, and finally washed with TE buffer. The DNA of bacteria in the cell plugs was separated on 1.0% agarose gels by electrophoresis with a CHEF Mapper system (Bio-Rad, USA) in the 0.5×TBE buffer. The electrophoresis was performed at 6 V/cm and 14°C. The pulse time increased from 1 to 20 s during 18 h run. DNA-PFGE marker (Bio-Rad, USA) was used as the size marker.

### Verification of the Absence of Linear Plasmid pBSSB1 in Δ*fis* by PCR and Southern-blot

To verify the absence of linear plasmid pBSSB1 in Δ*fis*, a pair of primers Pa(23173) and Pb(23750) (Table 2), which was designed according to the sequence of pBSSB1 reported previously [8], was used to amplify the corresponding fragments in order to investigate whether the linear plasmid pBSSB1 was absent in the mutant strain Δ*fis* of *S.* Typhi. In addition, the separated DNA fragments from PFGE was transferred onto the nitrocellulose membrane and subjected to Southern-blot with the biotin-labeled DNA fragments as the probe.

### Complementary Expression of *fis* in Δ*fis*

Primers P-*fis*-A and P-*fis*-B (Table 2), specific to upstream and downstream regions of the gene *fis* were used to amplify a promoterless *fis* gene with *pfu* DNA polymerase (Fermentas). An *Nco* I site and a *Sal* I site were added to the 5′-termini of primers P-*fis*-A and P-*fis*-B, respectively. An approximately 297 bp amplicon was inserted into the *Nco* I and *Sal* I sites of the expression vector pBAD/gIII (Invitrogen) to form the recombinant plasmid (pBAD*fis*). The positive plasmid pBAD*fis* was verified by digestion with *Nco* I and *Sal* I and sequence analysis. The Δ*fis* was transformed with pBAD*fis* and designated as Δ*fis*(pBAD*fis*). As a control, the Δ*fis* was also transformed with the empty vector pBAD/gIII and designated as Δ*fis*(pBAD). Expression of *fis* in Δ*fis*(pBAD*fis*) was induced by L-arabinose (0.2% wt/vol).

### Insertion of Kanamycin Cassette within pBSSB1

A kanamycin resistance gene was inserted within pBSSB1 between ORF001 and ORF002 using the lambda Red recombinase (one-step method) as described by Datsenko and Wanner [16]. First, the Red helper plasmid pKD46 was isolated and transformed into wild type *S*. Typhi GIFU10007 by electroporation (2.5 kV, 600 ohms, 25 µF; Bio-Rad Gene Pulser). Then, the kanamycin resistance gene was amplified with the primers P-Kana-A and P-Kana-B (Table 2) using plasmid pET-28a-c(+) DNA as a template. PCR-amplified DNA was precipitated and re-suspended in 10 µl of nuclease-free water. Re-suspended DNA was transformed by electroporation into *S*. Typhi GIFU10007 cells containing pKD46 plasmid as described above. Transformed cells were recovered for 1 h at 37°C in 800 µl of SOC, and then plated onto LB medium supplemented with 25 µg/ml kanamycin. Finally, the insertion of kanamycin cassette into pBSSB1 was confirmed by PCR with the primers ORF1-A and Kana-B (Table 2). The pBSSB1 plasmid with the kanamycin resistance gene insert was designated as pBSSB2, and *S*. Typhi GIFU10007 harboring pBSSB2 was designated as *S*. Typhi GIFU10007-1.

### Transformation of Δ*fis*, Δ*fis*(pBAD) and Δ*fis*(pBAD*fis*) with pBSSB2

The pBSSB2 plasmid was isolated from *S*. Typhi GIFU10007-1 using an alkaline lysis method originally described by Kado and Liu [17]. The quality and quantity of the extracted pBSSB2 DNA were tested by electrophoresis and an ND-1000 Spectrophotometer (NanoDrop Technologies, Wilmington, USA) respectively. Then, Δ*fis*(pBAD*fis*), Δ*fis*(pBAD) and Δ*fis* was transformed with the pBSSB2 DNA by electroporation (2.5 kV, 600 ohms, 25 µF; Bio-Rad Gene Pulser). Transformed cells were screened on the LB medium supplemented with 25 µg/ml kanamycin. Transformation of Δ*fis* or Δ*fis*(pBAD) or Δ*fis*(pBAD*fis*) with pBSSB2 was finally confirmed by PCR with primers Pa(23173) and Pb(23750) (Table 2).

### Motility Assay

Bacteria were cultured overnight at 37°C in LB broth. Each 4 µl of culture was inoculated into the centre of a 0.3% semisolid LB agar plate cntaining L-arabinose (0.2% wt/vol). The plates were incubated at 37°C for 10 hours and motility was assessed qualitatively by examining the diameter of circular swimming which was formed by the growing motile bacterial cells.

### Two-dimensional Polyacrylamide Gel Electrophoresis and Mass Spectrometry Analysis

Bacterial proteins were extracted from wild-type and *fis* mutant strain strains. The proteins were firstly separated by isoelectrofocusing electrophoresis and then subjected to SDS-PAGE electrophoresis (Bio-rad). After staining with Coomassie Brilliant Blue G-250, differential expression of bacterial proteins between the wild-type and *fis* mutant strains were detected and analyzed by Mass spectrometry.

## Results

### The Loss of Linear Plasmid pBSSB1 in Δ*fis* of *S.* Typhi

**Figure 1:**
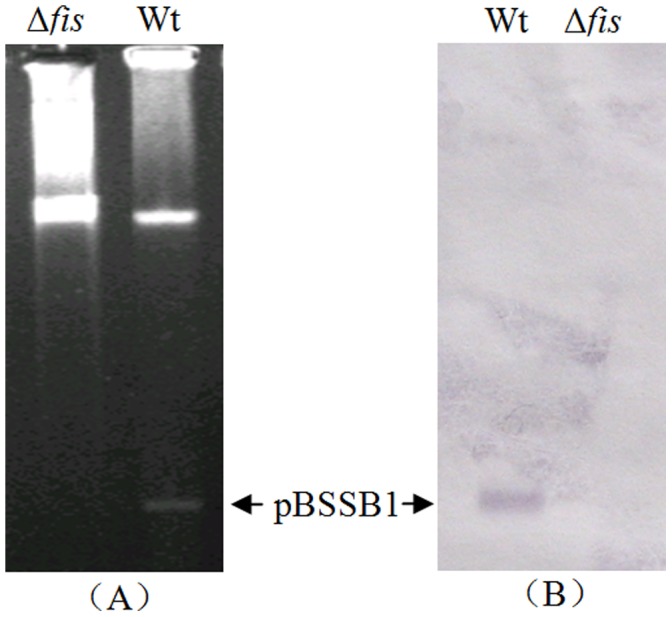
Identification of linear plasmid pBSSB1 by PFGE (A) and Southern-blot (B).

The DNA of bacteria in the agarose, which was not digested by restriction enzymes, was separated by PFGE. As shown in Figure 1(A), two DNA bands were stained and the upper band may be the chromosomal DNA while the lower band is the linear plasmid pBSSB1 DNA. This result indicates that the linear plasmid pBSSB1 was lost in the Δ*fis* of *S.* Typhi. To better verify the loss of the linear pBSSB1 plasmid, we used biotin labeled DNA fragment which was amplified by a pair of specific primers designed according to the sequence of pBSSB1 plasmid as probes to hybridize the DNA bands from PFGE by Southern blot (figure 1(B)). In addition, a specific DNA fragment of pBSSB1 can not detected by PCR with primers designed according to the pBSSB1 DNA sequence (data not shown). Moreover, the pBSSB2 plasmid DNA can only be transformed into the complementary strain Δ*fis*(pBAD*fis*) while the Δ*fis* and Δ*fis*(pBAD) cannot host the plasmid pBSSB2 DNA. These results show that Fis is essential for the stability of this linear plasmid in *S.* Typhi.

### Fis Affects the Motility of Δ*fis*

**Figure 2:**
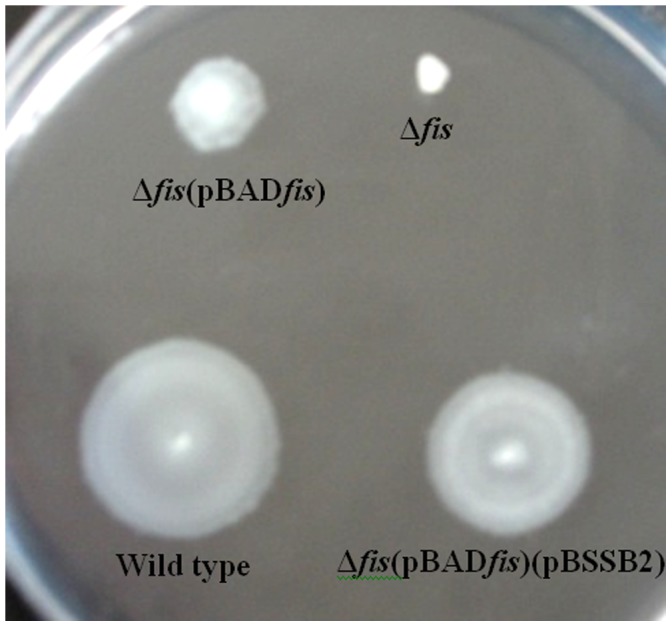
Effect of Fis on the motility of *S.* Typhi GIFU10007.

**Figure 3:**
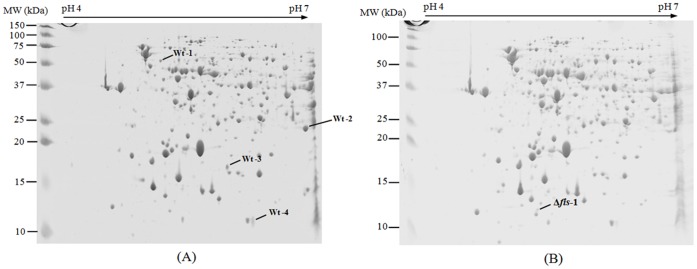
Two-dimensional polyacrylamide gel electrophoresis profile of wild-type strain (A) and Δ*fis* (B).

As shown in Figure 2, the motility of Δ*fis* was greatly decreased in comparison with the wild-type parental strain. However, motility was restored, to some extent in the complemented strains Δ*fis*(pBAD*fis*) and Δ*fis*(pBAD*fis*)(pBSSB2). In addition, the bacterial proteins of Δ*fis* of *S*. Typhi were compared with those of wild-type strain by two-dimensional polyacrylamide gel electrophoresis and mass spectrometry analysis. As shown in Figure 3, five bacterial proteins (Wt-1, Wt-2, Wt-3, Wt-4 and Δ*fis*-1), whose expression was obviously different between the mutant strain Δ*fis* and wild-type strain, were found by two-dimensional electrophoresis analysis. Among these bacterial proteins, the flagellin (Wt-1) encoded by *fljB* ^z66^ gene was confirmed to be absent in the mutant strain Δ*fis* by mass spectrometry analysis. These results demonstrate that Fis may affect the bacterial motility due to the loss of linear plasmid pBSSB1 in Δ*fis* of *S*. Typhi GIFU10007.

## Discussion

Although linear plasmids are relatively common in bacterial species such as *Streptomyces* and *Borrelia*, pBSSB1 is the first non-bacteriophage-related linear plasmid to be described in the Enterobacteriaceae that contains no detectable homology sequence of bacteriophage [8]. However, little is known about the replication of this linear plasmid. Fis is a very important small nucleotide-associated protein which plays a role in affecting the bacterial chromosome structure and the initiation of DNA replication [12]. In this study, the fact that pBSSB1 disappeared in the mutant strain Δ*fis* is a significant observation. This means that Fis is essential for the stability of the linear plasmid pBSSB1. There are reports to show that either the formation of multicopy plasmid dimers or the associated reduction in copy number leads to the instability of the plasmid [18], [19]. The global regulator Fis is essential for the stable maintenance of plasmid ColE1 through binding to cer of ColE1in a sequence-specific manner [20]. ColE1-like plasmids are less stable in *fis* mutant hosts and it is conceivable that instability caused by the mutation is due to altered Fis binding site [21]. Therefore, Fis may influence the stability of pBSSB1 plasmid by affecting a specific gene. However, there was no obvious differential expression of genes contributing to DNA replication found by the microarray and proteomic analysis between the wild-type and Δ*fis* (data not shown).

Changes in GC skew, which was reported to be associated with the origin of replication on plasmids and bacterial chromosomes, were previously used to predict the internal origin of bi-directional linear replication [22], [23]. It was reported that the change in GC skew ((G-C)/(G+C)) is present in the middle of pBSSB1 and this region may be the origin of the replication [8]. Therefore, it is speculated that the replication of pBSSB1 is initiated from the middle and prolonged bi-directionally and Fis may be essential for the initiation of replication of this plasmid. In addition, it has been suggested that pBSSB1 may possess terminal protein (Tp) covalently bound to the 5′ end of the DNA, which is very similar to linear plasmids from *Streptomyces* [8]. Many linear plasmids are replicated bi-directionally from an internal origin, which leaves single-stranded gaps of 250-300 nt at the 3′ ends, and these gaps are proposed to be patched by Tp-primed DNA synthesis [24], [25]. Therefore, Fis may also affect the replication of pBSSB1 through the regulation on the Tp of this plasmid. All these hypotheses need further experiments to clarify.

Previous studies showed that *S*. Typhi z66 positive strain is a biphasic *Salmonella serovar*, harbouring the *fliC* gene in the chromosome and *fljB* ^z66^ gene in the pBSSB1 plasmid [8], [14]. FljA^z66^, which is encoded by *fljA* ^z66^ gene located downstream of *fljB* ^z66^ gene, can inhibit the expression of *fliC* similar to most biphasic *Salmonella* [9], [15]. In this study, the loss of plasmid pBSSB1 in Δ*fis* may relieve the repression of FljA^z66^ on the expression of *fliC*. Previous study showed that the expression of the ﬂagellar genes selected from the early (*ﬂhD*), middle (*ﬂiA*) and late (*ﬂiC*) stages of ﬂagellar biosynthesis was strongly repressed in the *fis* mutant [3]. In the present study, we also found that many genes contributing to ﬂagellar biosynthesis and motility were strongly down regulated in Δ*fis* by microarray analysis of the differential expression between the wild-type and Δ*fis* (data not shown). Therefore, Fis might affect the bacterial motility due to the loss of pBSSB1 plasmid and the decreased expression of *fliC* in Δ*fis* of *S*. Typhi.

In summary, this study is the first to demonstrate that Fis is essential for the stability of pBSSB1 and affects the motility of *S.* Typhi.
